# Chloral in Midwifery

**Published:** 1871-03

**Authors:** 


Chloral in Midwifery.—Dr. Lambert, late House-Surgeon to the Edinburgh Maternity Hospital (*Edinburgh Medical Journal*), arrives at the subjoined conclusion, in regard to the administration of chloral in cases of labor:

Chloral is an agent of great value in relief of pain during parturition. It is demonstrated that a labor can be conducted from its commencement to its termination, without any consciousness on the part of the patient under the sole influence of chloral. The exhibition of chloral in nowise interferes with the exhibition of chloroform. The proper mode of administering chloral is in fractional doses of 15 grains every quarter of an hour until some effect is produced; and according to the nature of that effect, the further administration is to be regulated. Some patients will require doses of one drachm; and it is better to produce an anæsthetic effect by three drachms given in the space of two hours, than by one drachm given singly.

This agent rather promotes uterine contraction, by suspending all reflex actions which tend to counteract the incitability of the centres of organic motion.

Labors under chloral will probably be found to be of shorter duration than when natural. The general conditions under which chloral is to be administered are the same as those which regulate the administration of chloroform, and the rules laid down by the late Sir James Y. Simpson, M.D., in conneetion with this subject must be rigidly adhered to.

